# Comparative Analysis of the Metabolomic Profile of Honeysuckle *Lonicera caerulea* L. from Four Eurasian Regions by Using HPLC-ESI-MS and ESI-MS/MS Analysis

**DOI:** 10.3390/molecules30183761

**Published:** 2025-09-16

**Authors:** Mayya P. Razgonova, Muhammad Amjad Nawaz, Elena A. Rusakova, Andrey S. Sabitov, Nadezhda G. Tikhonova, Kirill S. Golokhvast

**Affiliations:** 1 N.I. Vavilov All-Russian Institute of Plant Genetic Resources, B. Morskaya 42-44, Saint-Petersburg 190000, Russia; andrsabitov@rambler.ru (A.S.S.); n.g.tikhonova@vir.nw.ru (N.G.T.); 2 Advanced Engineering School, “Institute of Biotechnology, Bioengineering and Food Systems”, Far Eastern Federal University, Fr. Russian, pos. Ajax, 10, Vladivostok 690922, Russia; 3 Advanced Engineering School of Agrobiotechnology, National Research Tomsk State University, Lenin Ave, 36, Tomsk 634050, Russia; golokhvast@sfsca.ru; 4 Laboratory for Research and Application of Supercritical Fluid Technologies in Agro-Food Biotechnology, National Research Tomsk State University, Lenin Ave, 36, Tomsk 634050, Russia; 5 FSBSI Kamchatsky Scientific Research Institute of Agriculture, Centralnaya, 4, Sosnovka 684033, Russia; rubusarcticus@mail.ru; 6 Siberian Federal Scientific Centre of Agrobiotechnology, Centralnaya, Presidium, Krasnoobsk 633501, Russia

**Keywords:** honeysuckle berries, *Lonicera caerulea*, metabolomics, polyphenols, tandem mass spectrometry

## Abstract

Blue honeysuckle (*Lonicera caerulea*) is widespread across the Eurasian continent, mainly in northern latitudes. Its berries are a rich source of biologically active compounds. In this study, plant samples collected in four regions of Russia separated by more than 10,000 km were examined in detail: St. Petersburg, Kamchatka, Magadan and the Far East (Vladivostok). The study was unique in that it covered almost the entire Eurasian continent in northern latitude, which had not been previously presented in other scientific studies. The study revealed the presence of 110 polyphenols and 34 compounds belonging to other chemical groups. In particular, honeysuckle berries were rich in polyphenols, including flavonoids, flavanones, flavanols, flavan-3-ols, anthocyanins, stilbenes, and lignans. The method of tandem mass spectrometry was used to identify biologically active substances from the extracts, which allows obtaining fairly accurate results. The metabolomic composition of *L. caerulea* berries originating from Kamchatka and Magadan showed the greatest diversity of polyphenols, which is associated with special northern climatic conditions and associated stress factors for plants. The results we obtained provide new data on the composition of the honeysuckle berry metabolome. The wealth of biologically active substances in blue honeysuckle berries can be very interestingly used in the development of both biologically active additives for pharmaceutical use and for the development of functional and specialized nutrition products for various population groups.

## 1. Introduction

**Figure 1:**
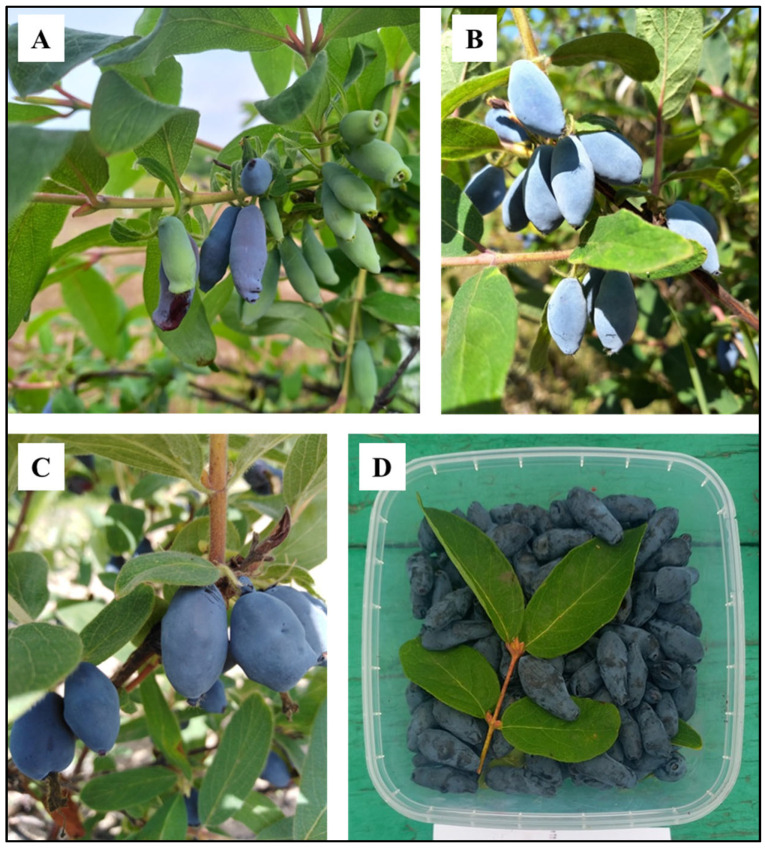
A representative figure of *L. caerulea* ssp. *kamtschatica* samples used for metabolome analysis. (**A**) Ripening of berries of *L. caerulea* ssp*. kamtschatica* growing in the N.I. Vavilov All-Russian Institute of Plant Genetic Resources, Kamchatka; (**B**) wild *L. caerulea* spp. *kamtschatica* Atlant; (**C**) wild *L. caerulea* spp. *kamtschatica* Darinka; (**D**) wild *L. caerulea* spp. *kamtschatica* Elena (Photos by E. Rusakova).

Blue honeysuckle (*Lonicera caerulea* L.) and *Lonicera caerulea* ssp. Kamtschatica, belonging to the Caprifoliaceae family (Figure 1), are widespread across the Eurasian continent, mainly in the northern parts, and come from high mountainous or humid regions. *Lonicera caerulea* L. is a well-known plant in China, North America, and Russia [1]. It is commonly known as honeysuckle berry (refers to the Japanese type of blue honeysuckle) or honeysuckle (Russian and Kuril varieties of blue honeysuckle). The plant is found in the wild in the forests of Europe, the Far East, Kamchatka, and along the entire coast of the Sea of Okhotsk, mainly in mountainous and lowland humid regions [2]. The Japanese Ainu aborigines considered honeysuckle berries to be the “elixir of life”, and on the island of Hokkaido, juice made from the fruits is sold as a “remedy for eternal youth and longevity” [3]. In recent years, it has become widely grown in Europe, in countries such as Poland, Slovenia, the Czech Republic, and Slovakia [4], due to the high content of vitamins and various biologically active substances in the berries [5].

The genus *Lonicera* consists of more than 200 species [6]. Currently, the most commonly planted honeysuckle berries are those originating from Russia, Japan, Canada, and Poland. The most popular and easy-to-grow varieties in Russia are tundra, borealis, indigo gem, blue lightning, and kamchatka [7]. Owing to their adaptations to the environmental conditions of these regions, they exhibit higher low-temperature tolerance; the honeysuckle plants can withstand up to −40 °C, whereas the flowers can withstand up to −7 °C. Moreover, they are least affected by changes in soil pH, the presence of pests, or diseases [4,7]. The honeysuckle berries contain more than 85% moisture, followed by fiber (~8%), crude protein (~2%), fats (>0.1%), and carbohydrates (~0.9%). More than 180 compounds have been reported in the fruits of honeysuckle. Major compounds are amino acids and their derivatives, vitamins, minerals, sugars (e.g., fructose, glucose, sucrose, saccharose, sorbitol, and others), phenolic acids, flavonoids, terpenoids, fatty acids, organic acids, and carotenoids [4,5]. Based on the presence of a range of vitamins, several studies referred to it as a “super fruit” [6,7].

Regular consumption of blue honeysuckle berries has been associated with health benefits such as the prevention of chronic diseases, diabetes, and cardiovascular disease [8]. The predominant phenolic compound present in blue honeysuckle berries is cyanidin-3-*O*-glucoside, the most abundant anthocyanin in nature. An earlier study showed that cyanidin-3-*O*-glucoside isolated from blue honeysuckle enhances insulin production and is responsible for hepatoprotective effects through the inhibition of reactive oxygen species and activation of antioxidant mechanisms [9]. Generally, anthocyanins can improve metabolism by activating adenosine monophosphate-activated protein kinase (AMPK). In addition to health benefits, these anthocyanins and flavonols generate the vibrant blue color of the berries [10]. The metabolomic composition of honeysuckle fruits also suggests other nutritional benefits such as anti-obesity, strong anti-inflammatory, anti-diabetic, anti-tumor, and cardioprotective properties, as well as several other protective effects on the liver and thyroid [7,11,12]. It has also been shown that a number of compounds present in honeysuckle plants may be beneficial for plant–environment interactions. For example, a study showed that metabolites related to alkaloid biosynthesis, tricarboxylic acid cycle, phenylpropanoid biosynthesis, and terpenoid biosynthesis accumulated in salt-stressed plants compared to control samples [13]. Similarly, honeysuckle plants increased phenolic acid content under salt stress conditions [14]. It has been repeatedly emphasized that understanding the metabolome is important in natural and stress conditions for both plant growth and development and for applications in healthy nutrition and natural vitamin supplementation. However, as noted in our recent works, honeysuckle berries collected in different locations vary in their primary and secondary metabolite contents [15,16,17]. Considering that honeysuckle is grown over a wide geographical range across the Eurasian continent, it is necessary to further study the metabolomic composition of different varieties grown in different locations. This information is useful for both the discovery of new compounds and for understanding the impact of honeysuckle cultivation in different locations so that appropriate strategies can be adapted to collect the desired metabolomic content for medicinal purposes. In this study, we report the metabolome composition of *L. caerulea* berry extracts from four geographical areas separated by more than 7000 km using HPLC-ESI-MS and ESI-MS/MS analyses.

## 2. Results and Discussion

### 2.1. Optimization of HPLC Conditions

The HPLC conditions were optimized to obtain maximal resolution and signal within a minimal run time. Various chromatographic conditions such as mobile phase composition, injection volume, flow rate, column temperature, and gradient program were studied and optimized for the separation of polyphenol compounds. Different mobile phase compositions (ethanol–water, ethanol–0.1% (*v*/*v*) formic acid aqueous solution, acetonitrile–water, and acetonitrile–0.1% (*v*/*v*) formic acid aqueous solution) were tested in the gradient program at 0.25 mL/flow rate. A mobile phase composed of 0.1% (*v*/*v*) formic acid aqueous solution (A) and acetonitrile (B) at a 0.25 mL/min flow rate and 50 °C column temperature was found optimal for the resolution of the maximum number of peaks in extracts of *L. caerulea* within 60 min.

### 2.2. Tentative Identification of Compounds from the Extracts of Honeysuckle Berries

We were able to identify one hundred forty-four chemical compounds from extracts of honeysuckle berries: one hundred ten chemical compounds from the polyphenol group and thirty-four chemical compounds from other chemical groups. All identified polyphenols and other compounds, along with molecular formulas and MS/MS data for *L. caerulea*, are summarized in Appendix A Table A1. The polyphenols detected in our study were further categorized as flavan-3-ols, flavones, flavanols, tannins, phenolic acids, lignans, coumarins, stilbenes, anthocyanidins, etc. These numbers indicate that *L. caerulea* berries are rich in flavonoids. Moreover, the presence of a higher number of anthocyanins indicates their roles in the color formation of the berries.

Anthocyanins are major color-forming pigments in honeysuckle berries [5,8,13,18,19]. The extract analyses of berries of both the *L. caerulea* varieties and six wild type accessions by employing HPLC-ESI-MS and ESI-MS/MS resulted in the detection of nineteen anthocyanins. A notable result was that the extracts of the berries of *L. caerulea* varieties contained delphinidin 3-*O*-glucoside, delphinidin 3-*O*-rutinoside, delphinidin 3-*O-β-D*-sambubioside, cyanidin 3-*O*-rutinoside, peonidin 3-*O*-rutinoside, delphinidin 3-acetylglucoside, cyanidin 3,5-*O*-diglucoside, etc. However, cyanidins were absent from the extracts of the berries of *L. caerulea*.

**Figure 2:**
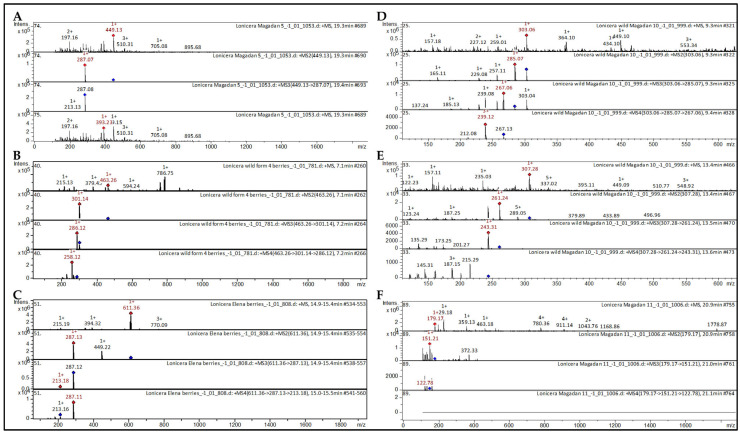
CID spectrum of selected compounds. (**A**) Cyanidin 3-*O*-glucoside from extracts from berries of *L. caerulea*, *m*/*z* 449.13. At the top is an MS scan in the range of 100–1700 *m*/*z*, at the bottom are fragmentation spectra (from **top** to **bottom**): MS2 of the protonated cyanidin 3-*O*-glucoside ion (449.13 *m*/*z*, red diamond), MS3 of the fragment 449.13 → 287.07 *m*/*z*, and MS4 of the fragment 303.06 → 285.07 → 213.13 *m*/*z*. (**B**) CID spectrum of peonidin 3-*O*-glucoside from extracts from berries of *L. caerulea*, *m*/*z* 463.26. At the top is an MS scan in the range of 100–1700 *m*/*z*, at the bottom are fragmentation spectra (from **top** to **bottom**): MS2 of the protonated peonidin 3-*O*-glucoside ion (463.26 *m*/*z*, red diamond), MS3 of the fragment 463.26 → 301.14 *m*/*z*, and MS4 of the fragment 463.26 → 301.14 →286.12 *m*/*z*. (**C**) CID spectrum of cyanidin 3,5-*O*-diglucoside from extracts from berries of *L. caerulea*, *m*/*z* 611.36. At the top is an MS scan in the range of 100–1700 *m*/*z*, at the bottom are fragmentation spectra (from **top** to **bottom**): MS2 of the protonated cyanidin 3,5-*O*-diglucoside ion (611.36 *m*/*z*, red diamond), MS3 of the fragment 611.36 → 287.13 *m*/*z*, and MS4 of the fragment 611.36 → 287.13 → 213.18 *m*/*z*. (**D**) CID spectrum of quercetin from extracts from berries of *L. caerulea*, *m*/*z* 303.06. At the top is an MS scan in the range of 100–1700 *m*/*z*, at the bottom are fragmentation spectra (from **top** to **bottom**): MS2 of the protonated quercetin ion (303.06 *m*/*z*, red diamond), MS3 of the fragment 303.06 → 285.07 *m*/*z*, and MS4 of the fragment 303.06 → 285.07 → 267.06 *m*/*z*. (**E**) CID spectrum of gallocatechin from extracts from berries of *L. caerulea*, *m*/*z* 308.28. At the top is an MS scan in the range of 100–1700 *m*/*z*, at the bottom are fragmentation spectra (from **top** to **bottom**): MS2 of the protonated gallocatechin ion (307.28 *m*/*z*, red diamond), MS3 of the fragment 307.28 → 261.24 *m*/*z*, and MS4 of the fragment 307.28 → 261.24 → 243.31 *m*/*z*. (**F**) CID spectrum of esculetin from extracts from berries of *L. caerulea*, *m*/*z* 179.17. At the top is an MS scan in the range of 100–1700 *m*/*z*, at the bottom are fragmentation spectra (from **top** to **bottom**): MS2 of the protonated esculetin ion (179.17 *m*/*z*, red diamond), MS3 of the fragment 179.17 → 151.21 *m*/*z*, and MS4 of the fragment 179.17 → 151.21 →122.78 *m*/*z*.

Three tentatively identified CID spectra (collision-induced spectra) of anthocyanins in *L. caerulea* extracts are presented below (Figure 2A). Anthocyanin cyanidin 3-*O*-glucoside was found in the extracts from berries of *L. caerulea* (Figure 2A).

The [M + H]^+^ ion produced one fragment ion with *m*/*z* 287.07. The fragment ion with *m*/*z* 287.07 produced one characteristic daughter ion with *m*/*z* 213.13. Mass spectrometry of cyanidin 3-*O*-glucoside is presented in detail in scientific studies on *Triticum aestivum* [20]; *Black soybean* [21]; *Glycine soja* [22]; *Red Kiwifruit* [23]; *black currant*, *gooseberry*, *chokeberry*, *elderberry*, *red currant* [24]; *Ribes magellanicum* [25]; *Rubus ulmifolius* [26]; *Berberis ilicifolia*; *Berberis empetrifolia*; *Ribes magellanicum*; *Ribes cucullatum*; *Myrteola nummalaria*; *Gaultheria mucronata*; *Gaultheria antarctica*; *Rubus geoides*; *Fuchsia magellanica* [27]; *Berberis microphylla* [28]; *Fragaria vesca* [29] extracts.

**Figure 3:**
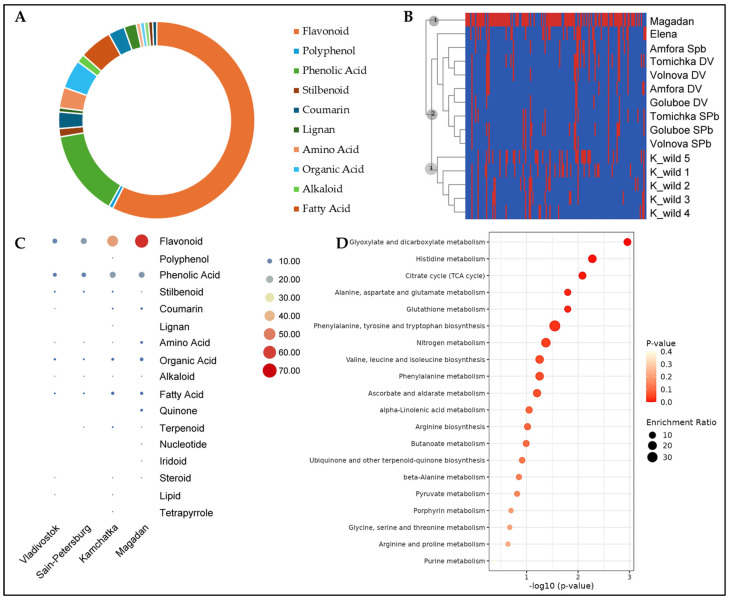
**Metabolite profile of the berries from honeysuckle varieties and wild type from different regions.** (**A**) Pie chart of the number of compounds classified in each compound class. (**B**) Heatmap based on presence/absence of metabolite in the studied honeysuckle varieties/wild type. (**C**) Number of compounds from each class detected in each location. (**D**) Scatter-plot of KEGG pathways to which the metabolites were enriched. In panel B, K_wild = wild type from Kamchatka, DV = Far east - Vladivostok, SPb = Saint Petersburg.

Anthocyanin peonidin 3-*O*-glucoside was found in the extracts from berries of *L. caerulea* (Figure 3B). The [M + H]^+^ ion produced one fragment ion with *m*/*z* 301.14. The fragment ion with *m*/*z* 301.14 produced one characteristic daughter ion with *m*/*z* 286.12. The fragment ion with *m*/*z* 286.12 produced one characteristic daughter ion with *m*/*z* 258.12. Similar mass spectrometry of peonidin 3-*O*-glucoside is indicated in scientific studies devoted to *Vitis vinifera* [30,31]; Vines [32]; *Vitis labrusca* [33]; *Vitis vinifera*; *Vitis rupestris* [34]; *Fragaria vesca* [35]; *Triticum aestivum* [36]; *Vigna sinensis* [37]; *Vigna unguiculata* [38] extracts.

Anthocyanin cyanidin 3,5-*O*-diglucoside was found in the extracts from berries of *L. caerulea* (Figure 2C). The [M + H]^+^ ion produced one fragment ion with *m*/*z* 287.13. The fragment ion with *m*/*z* 287.13 produced one characteristic daughter ion with *m*/*z* 213.18. Similar mass spectrometry of cyanidin 3,5-*O*-diglucoside is indicated in scientific studies devoted to honey [39]; grape [40]; *Vitis vinifera* [31]; *Vitis labrusca* [33]; *Vitis vinifera*; *Vitis rupestris* [34]; *Muscadine pomace* [41]; *Berberis microphylla* [28]; elderberry, red currant [24]; rapeseed petals [42] extracts.

Several tentatively identified CID spectra (collision-induced spectra) of chemical compounds in *L. caerulea* extracts are presented below (Figure 3). Among the identified metabolites, flavanol quercetin was found in the extracts from berries of *L. caerulea* (Figure 2D). The [M + H]^+^ ion produced four fragment ions with *m*/*z* 285.07, *m*/*z* 257.11, *m*/*z* 229.08, and *m*/*z* 165.11. The fragment ion with *m*/*z* 285.07 produced four characteristic daughter ions with *m*/*z* 267.06, *m*/*z* 239.08, *m*/*z* 185.13, and *m*/*z* 137.24. The fragment ion with *m*/*z* 267.06 produced two daughter ions with *m*/*z* 239.12 and *m*/*z* 212.08. Mass spectrometry of quercetin is presented in detail in scientific studies on *Inula graveolens* [43]; *Juglans mandshurica* [44]; black soja [21]; *Rhus coriaria* [45]; potato leaves [46] extracts.

The flavan-3-ol gallocatechin was also discovered in the extracts from berries of *L. caerulea* (Berry picking place –Magadan) (Figure 2E). The [M + H]^+^ ion produced four fragment ions with *m*/*z* 289.05, *m*/*z* 261.24, *m*/*z* 187.25, and *m*/*z* 123.24. The fragment ion with *m*/*z* 261.24 produced four characteristic daughter ions with *m*/*z* 243.31, *m*/*z* 201.27, *m*/*z* 173.25, and *m*/*z* 135.29. The fragment ion with *m*/*z* 243.31 produced three daughter ions with *m*/*z* 215.29, *m*/*z* 187.15, and *m*/*z* 145.31. Mass spectrometry of gallocatechin is presented in detail in scientific studies on *Carpinus betulus* [47]; Solanaceae [48]; Rhododendron [49]; *Ribes meyeri* [50]; *Licania ridigna* [51]; *G. linguiforme* [52]; *Senna singueana* [53]; Embelia [54] extracts.

The hydroxycoumarin esculetin was also discovered in the extracts from berries of *L. caerulea* (Berry picking place—Magadan) (Figure 2F). The [M + H]^+^ ion produced one fragment ion with *m*/*z* 151.21. The fragment ion with *m*/*z* 151.21 produced one characteristic daughter ion with *m*/*z* 122.78. Mass spectrometry of esculetin is presented in detail in scientific studies on S*alvia* spp. [55]; *Artemisia annua* [56]; *Actinidia* [57]; *Rhinacanthus nasutus* [58]; *Basilic*; *Rosemary*; *Salvia*; *Thymus vulgaris* [59]; *Dryopteris fragrans* [60] extracts.

### 2.3. Metabolite Profile of Studied Honeysuckle Berries

Overall, the metabolites detected in our study belonged to 40 compound classes. The highest number of metabolites was flavonols (28), followed by anthocyanins (21), phenolic acids (19), flavone (17), and flavan-3-ols (12) (Figure 3A). Based on the presence/absence of compounds, the studied honeysuckle varieties/wild types were clustered into three groups. Cluster 1 consisted of the wild-type honeysuckle from the Magadan region, whereas the remaining varieties/wild types were grouped into two clusters. Cluster 2 consisted of all the cultivated varieties of honeysuckle included in the study. In cluster 2, we noticed that only the variety Elena formed a separate branch, while the rest tended to group together. Those cultivated in St. Petersburg, i.e., Tomichka, Goluboe, and Volnova, clustered together, except for Amfora. Cluster 3 consisted of all the wild-type honeysuckle cultivated in the Kamchatka region (Figure 3B). In terms of the number of compounds detected in honeysuckle from each location, the Magadan region had the highest number of flavonoids, phenolic acids, coumarins, amino acids, fatty acids, and quinones (Figure 3C). The detected metabolites were enriched in 20 KEGG pathways (Figure 3D).

**Figure 4:**
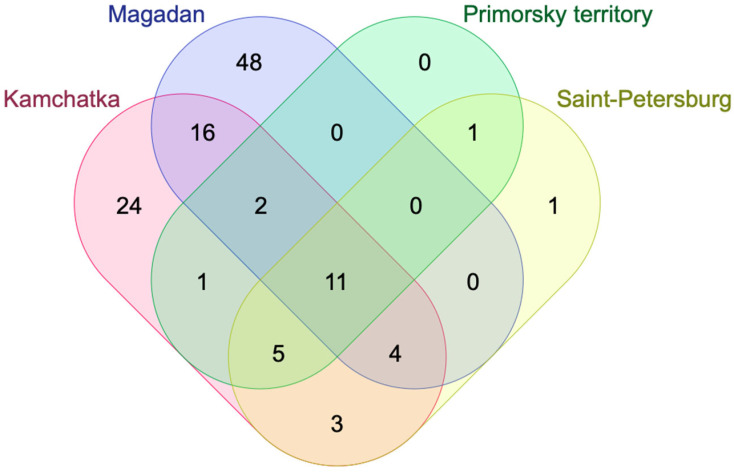
The Venn diagram showing the similarities and differences between *L. caerulea* collected in four geographical areas (Saint Petersburg, Kamchatka, Magadan, Primorsky territory).

Figure 4 shows the similarities and differences between *L. caerulea* collected in four geographical areas: Saint Petersburg, Kamchatka, Magadan, and Primorsky territory (Vladivostok).

To present the similarities and differences in bioactive substances in different variations of *L. caerulea*, we used the Jaccard index (Table 1). The Jaccard index, also known as the Jaccard similarity coefficient, is a statistic used to evaluate the similarity and diversity of sets of samples [61]. It showed that the highest degree of similarity is present between the varieties from Saint Petersburg and Kamchatka—0.3382.

Table 2 shows the distribution of the polyphenol groups in *L. caerulea* samples from four places of collection (Kamchatka, Magadan, Saint Petersburg, Primorsky territory).

These results highlight that the wild type collected from the Magadan region is richest in terms of the number of compounds, followed by Kamchatka. Moreover, the wild-type honeysuckle collected and grown in the Kamchatka region offers a similar profile in terms of detected compounds, whereas the cultivated varieties share common metabolite profiles. Since both Saint Petersburg and Vladivostok had common honeysuckle types and exhibited only one common metabolite, it can be understood that geographic location plays an important role in the metabolite profile of honeysuckle berries.

The metabolomic profiles of honeysuckle berries reveal significant geographical variation in polyphenolic composition, reflecting both genetic differences and environmental adaptations. Polyphenols, classified based on phenolic rings and functional groups, include flavonoids (flavanols, flavonols, flavones), phenolic acids, stilbenes, and lignans [62]. Our identification of 110 polyphenols from 40 structural classes confirms honeysuckle’s status as a rich source of bioactive compounds, with northern populations (Magadan, Kamchatka) showing particularly complex profiles. Flavonoids were the most found metabolites in the honeysuckle berry metabolite profiles, which is consistent with the geographic presence of the studied samples and their roles in UV protection and stress response [63,64]. The northern samples accumulated specialized forms like methylated quercetin derivatives and acylated anthocyanins, likely as adaptations to harsh climates [18,19]. This geographical pattern was quantified by Jaccard indices (Table 1), showing the lowest similarity (0.1477) between Magadan and Primorsky populations. Notably, cyanidin-3-*O*-glucoside (Figure 3) appeared exclusively in northern samples, suggesting differential regulation of flavonoid biosynthesis pathways [10,18].

**Figure 5:**
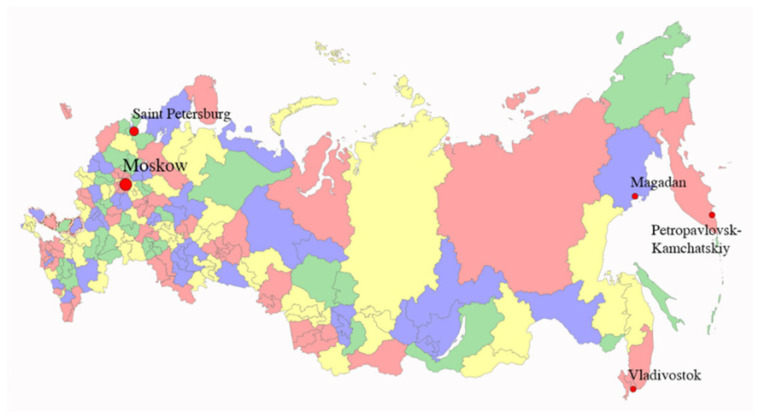
The map of the location of *L. caerulea* berry harvesting areas: collection of N.I. Vavilov All-Russian Institute of Plant Genetic Resources, St. Petersburg, and plantations by N.I. Vavilov All-Russian Institute of Plant Genetic Resources, Primorsky Territory, Kamchatka region, Magadan region.

Anthocyanins are key biomarkers of environmental adaptation. While delphinidin derivatives were largely found in Magadan, southern populations accumulated diglycosylated forms like cyanidin 3,5-*O*-diglucoside (Figure 5) [31,33]. These patterns align with known temperature effects on glycosylation [40,41] and validate honeysuckle’s traditional use for vascular health [9]. The vibrant berry coloration results from these anthocyanins [10], with mixtures showing greater bioactivity than individual compounds [65,66].

Phenolic acids showed distinct regional signatures. Chlorogenic acid was ubiquitous, but northern samples uniquely contained complex shikimate derivatives like feruloyl-*O*-p-coumaroyl-*O*-caffeoylshikimic acid—compounds associated with stress responses [13,14]. This supports findings that phenolic acid metabolism dynamically adjusts to environmental conditions [67,68]. Their antioxidant properties may contribute to observed health benefits [69,70,71].

The flavan-3-ol profile suggests significant nutraceutical potential. Northern samples contained oligomeric forms including (epi)gallocatechin-(epi)-catechin dimers, structurally similar to green tea catechins [72]. These compounds, particularly EGCG, show well-documented chemopreventive effects [73,74]. Molecular dynamics simulations have revealed their ability to bind phospholipid membranes, potentially enhancing bioavailability [75]. The ECG fraction may inhibit cancer cell invasion through MMP-2 suppression, though clinical efficacy depends on delivery methods [76].

Stilbenes and lignans showed localized distributions. Resveratrol appeared in Kamchatka, Magadan, Primorsky, and St. Petersburg samples. It has been previously reported that *Vitis amurensis* [77], Kamchatka berries, contained syringaresinol—a lignan also found in Schisandra chinensis [78]. These compounds contribute to honeysuckle’s traditional reputation as an “elixir of life” through anti-aging and hepatoprotective effects [4,5].

The therapeutic implications of these geographical differences are substantial. Northern berries’ combination of anthocyanins, oligomeric catechins, and complex phenolics creates synergistic conditions that may enhance bioavailability [79,80]. Formononetin was detected only in Magadan samples. This validates traditional preferences for northern varieties while suggesting modern cultivation strategies [7,15].

Most identified polyphenols can cross the blood–brain barrier [81], with antioxidant and anti-inflammatory activities that may protect against neurodegeneration [82,83]. Regular consumption could mitigate oxidative stress-linked disorders [84], though human studies remain limited. Overall, this study bridges traditional herbal usage knowledge and metabolomics. The Ainu’s “elixir of life” designation [3] finds support in the stress-induced metabolite complexity of northern varieties. Future work should couple these findings with transcriptomics to identify regulatory mechanisms [13] and optimize cultivation for bioactive content [4,7]. The demonstrated geographical variation underscores the importance of provenance in both research and commercial applications.

## 3. Materials and Methods

### 3.1. Plant Material

Eleven *L. caerulea* varieties and wild-type plants were studied. Wild form No 1, wild form No 2, wild form No 3, wild form No 4, wild form No 5, and variety “Elena” were collected and grown in Kamchatsky Scientific Research Institute, Kamchatka, Russia (N 43°6′34″, E 131°52′41″. Four varieties (Goluboe vereteno, Tomichka, Amfora, and Volnova) were collected and grown in N.I. Vavilov All-Russian Institute of Plant Genetic Resources, Primorsky Territory (N 53°11′, E 158°23′). These four varieties were also cultivated in N.I. Vavilov All-Russian Institute of Plant Genetic Resources, St.-Petersburg (Pushkin, N 59°42′51″, E 30°23′47″). One wild-type honeysuckle (Magadan) was collected in the Magadan region: the vicinity of Kolyma River (N = 59°4141′960; E = 151°16′17.620); Seymchansky district near the Kolyma River (N = 62°55′51.017; E = 151°16′17.620) (Figure 5).

Standard agronomic practices were followed for growing the accessions/varieties in their respective growing locations. The berries were harvested at the end of July 2023 from three-year-old plants (Figure 1). Triplicate samples were collected for each accession/variety. Care was taken to collect healthy, disease- and insect-free berries. The samples were washed with distilled water and stored at −80 °C until processed. All samples morphologically corresponded to the pharmacopeial standards of the State Pharmacopoeia of the Russian Federation. The berries of the Magadan wild type were collected, washed thrice using distilled water, frozen in liquid nitrogen, transported to the lab, and stored in −80 °C until further processed.

### 3.2. Chemicals and Reagents

All chemicals used in this study were of analytical grade. High-performance liquid chromatography (HPLC)-grade acetonitrile was purchased from Fisher Scientific (Southborough, UK). Mass spectrometry (MS)-grade formic acid was purchased from Sigma-Aldrich (Steinheim, Germany). Ultra-pure water was prepared by using a SIEMENS ULTRA clear (SIEMENS water technologies, Gunzburg, Germany).

### 3.3. Extraction

The fractional maceration technique was used to obtain highly concentrated extracts [85,86]. Aqueous ethanol (95% EtOH) was used for extraction. From 250 g of the berries, 50 g of berries of each variety were randomly selected for maceration. The total volume of the extractant (95% EtOH) was divided into 3 parts, and the berries of each studied species were successively infused in the first, second, and third parts. Infusion of each extract lasted for seven days in a dark room at room temperature. The extraction process for the berries of each studied species was carried out three times. The extract was filtered through Whatman paper.

### 3.4. Liquid Chromatography

High-performance liquid chromatography was performed using a Shimadzu LC-20 Prominence HPLC (Shimadzu, Kyoto, Japan) equipped with a UV sensor and a C18 silica reverse-phase column (4.6 × 150 mm, particle size: 2.7 μm) to perform the separation of multicomponent mixtures. The gradient elution program with two mobile phases (A, deionized water; B, acetonitrile with formic acid 0.1% *v*/*v*) was as follows: 0–2 min, 0% B; 2–50 min, 0–100% B; control washing 50–60 min 100% B. The entire HPLC analysis was performed with a UV–vis detector SPD- 20A (Shimadzu, Kyoto, Japan) at a wavelength of 230 nm for identification of compounds; the temperature was 40 °C, and the total flow rate was 0.25 mL min^−1^. The injection volume was 10 µL. Additionally, liquid chromatography was combined with a mass spectrometric ion trap to identify compounds.

### 3.5. Mass Spectrometry

Mass spectrometry analysis was performed on an ion trap amaZon SL (Bruker Daltonics, Bremen, Germany) equipped with an ESI source in positive and negative ion modes. The optimized parameters were obtained as follows: ionization source temperature—70 °C, gas flow—4 L/min, nebulizer gas (atomizer)—7.3 psi, capillary voltage—4500 V, end plate bend voltage—1500 V, fragmentary—280 V, collision energy—60 eV. An ion trap was used in the scan range *m/z* 100–1.700 for MS and MS/MS. The chemical constituents were identified by comparing their retention index, mass spectra, and MS fragmentation with an in-house self-built database (Biotechnology, Bioengineering and Food Systems Laboratory, Far-Eastern Federal University, Russia). The in-house self-built database is based on data from other spectroscopic techniques, such as nuclear magnetic resonance, ultraviolet spectroscopy, and MS, as well as data from the literature that is continuously updated and revised. The capture rate was one spectrum/s for MS and two spectra/s for MS/MS. Data acquisition was controlled by Windows software (Version 2.0) for Bruker Daltonics. All experiments were repeated three times. A four-stage ion separation mode (MS/MS mode) was implemented.

The detected putatively identified compounds were also enriched using MetaboAnalyst 6.0 (https://www.metaboanalyst.ca/MetaboAnalyst/home.xhtml) (accessed on 20 May 2025), for which scatter plot was generated. For KEGG pathway enrichment, standard parameters were used with Arabidopsis KEGG pathway library. For further analysis, the presence/absence data of the detected metabolites were grouped per location, i.e., all the varieties/wild types grown in one location were considered one group. The data was used to prepare a scatter plot and a heatmap with hierarchical clustering in TBtools Version 3.0 [87].

## 4. Conclusions

In this study, we report a comparative metabolomic profile of *L. caerulea* berries from four different provenances separated by more than 7000 km. We conclude that L. caerulea samples collected in the northern areas (Kamchatka and Magadan) have the highest variability in polyphenol composition compared to samples from areas with milder climates. Based on HPLC-ESI-MS and ESI-MS/MS analyses, we conclude that *L. caerulea* berries are rich in polyphenols (more than one hundred ten identified compounds), and the predominant classes are flavonoids, flavanones, flavonols, flavan-3-ols, and anthocyanins. In addition, about forty other chemical classes of metabolites including indole sesquiterpene alkaloids, iridoid glucosides, phenylpropanoid glucosides, amino acids and derivatives, nucleotides and derivatives, omega-hydroxy amino acids, etc., are the major components of *L. caerulea* berries.

## Figures and Tables

**Table 1:** Jaccard index for four places to collect *L. caerulea* (Kamchatka, Magadan, Saint Petersburg, Primorsky territory (Vladivostok)).

	Kamchatka (66)	Magadan (81)	Primorsky Territory (20)	Saint-Petersburg (25)
Kamchatka (66)		33 0.2895	19 0.2836	23 0.3382
Magadan (81)	33 0.2895		13 0.1477	15 0.1648
Primorsky territory (20)	19 0.2836	13 0.1477		17 0.6071
Saint-Petersburg (25)	23 0.3382	15 0.1648	17 0.6071	

**Table 2:** The distribution of the polyphenol groups in *L. caerulea* samples from four places of collection (Kamchatka, Magadan, Saint Petersburg, Primorsky territory).

Territory Name	Number of Detected Compounds	Compounds
Kamchatka; Magadan; Primorsky territory; Saint-Petersburg	11	Kaempferol; Herbacetin; *p*-Coumaroyl malonyldihexose; Ellagic acid; Resveratrol; Quercetin; (*Epi*)-afzelechin derivative; Hydroxy methoxy dimethylbenzoic acid; Delphinidin 3-*O*-*β*-D-sambubioside; Chlorogenic acid; Caffeoylquinic acid derivative
Kamchatka; Magadan; Primorsky territory	2	Fraxetin; Kaempferol 3-*O*-rutinoside
Kamchatka; Magadan; Saint-Petersburg	4	Epiafzelechin; (*Epi*)-catechin; Protocatechuic acid; Luteolin 7*-O*-glucoside
Kamchatka; Primorsky territory; Saint-Petersburg	5	Isorhamnetin 3-*O*-(6″-*O*-rhamnosyl-hexoside); Hydroxyferulic acid; Rhamnocitrin; Pinosylvin; Dihydroxy-tetramethoxy(iso)flavone
Kamchatka; Magadan	16	Delphinidin 3-acetylglucoside; Catechin; Neochlorogenic acid; Cyanidin-3-*O*-glucoside; Delphinidin; Cyanidin 3,5-*O*-diglucoside; Kaempferol glucosyl-rhamnoside; Kaempferol-3-*O*-hexoside; Cyanidin-3-*O*-*β*-galactoside; Diosmetin O-hexoside; Astragalin; Cyanidin-3-*O*-rutinoside; Myricetin; Kaempferol-3,7-Di-*O*-glucoside; Taxifolin-3-*O*-hexoside; Peonidin-3-O-glucoside
Kamchatka; Primorsky territory	1	Rutin
Kamchatka; Saint-Petersburg	3	Petunidin; 3-*O*-Hydroxydihydrocaffeoylquinic acid; (*Epi*)-catechin derivative 2
Primorsky territory; Saint-Petersburg	1	2,3,4,5,6-pentahydroxybenzoic acid
Kamchatka	24	Phloretin; 2,4,5,6-pentahydroxybenzoic acid; Kaempferol derivative; 4-Methylesculetin; (*epi*)-Catechin O-hexoside; Peonidin 3-*O*-rutinoside; *p*-Coumaroylquinic acid; Proanthocyanidin B-type; Di-*O-*galloyl-HHDP-glucose; Proanthocyanidin B1; Feruloyl-*O*-*p*-coumaroyl-*O*-caffeoylshikimic acid; Quercetin pentosyl hexoside hexoside; (*Epi*)-catechin derivative 3; 2′-Hydroxygenistein; (*Epi*)-catechin derivative 1; Syringaresinol; (*Epi*)gallocatechin-(*epi*)catechin dimer; Chrysoeriol *O-*diglucoside; Peonidin 3-*O*-(*6*-*O*-*p*-coumaroyl)glucoside; 3,4-Dihydroxyhydrocinnamic acid; Quercetin deoxyhexosyl deoxyhexosyl hexoside; Ferulic acid-*O*-hexoside derivative; Dicaffeoyl shiikimic acid
Magadan	48	Quercetin 3-(6-*O*-acetyl)glucoside; Delphinidin 3-*O*-[2-*O*-(β-xylosyl)-*β*-galactoside]; Nepetin; Esculetin; Quercetin 3-*O*-arabinoglucoside; (*Epi*)-Gallocatechin; Dimethylquercetin-3-*O*-dehexoside 7-*O*-diglucoside; Coriandrone; Lonicerin; Rhamnosyl-hexosyl-acyl-quercetin; Isorhamnetin acetyl galactoside; Isorhamnetin; Delphinidin 3-*O*-hexoside; Isorhamnetin acetylglucoside; Hesperitin; Prunetin; (-)-Epicatechin Gallate; Kaempferol *O*-acetyl hexosyl-rhamnoside; Caffeic acid isoprenyl ester; Quercetin 3-glucoside; Luteolin-*O*-hexoside; 4-Hydroxybenzoic acid; Cyanidin-3-*O*-hexoside; Quercetin 3-*O*-pentosyl hexoside; Luteolin; Luteolin 7-*O*-(*6*-*O*-rhamnosyl-hexoside); Hyperoside; Apigenin-7-*O*-*β*-*D*-glucopyranoside; Methylgallic acid; Ethyl protocatechuate; Luteolin 3′,7-*O*-diglucoside; Delphinidin 3-*O*-glucoside; Apigenin; Ellagic acid-*O-*hexoside; Gallocatechin; Jaceosidin; Delphinidin 3-*O*-(*6*-*O*-*p*-coumaroyl) glucoside; Quercetin 3-*O*-acetyl hexoside; Formononetin; Delphinidin 3-*O*-rutinoside; Delphinidin-3-*O*-(*6″*-*O*-acetyl)hexoside; Quercetin-3-*O*-rhamnoside derivative; 3,4-Dihydroxyhydrocinnamic acid; Quercetin-3-*O*-hexoside; Epigallocatechin gallate dimer; Delphinidin 3-*O*-*β*-galactoside; Petunidin 3-*O*-(*6*-*O*-acetyl)glucoside
Saint-Petersburg	1	Anthocyanidin [cyanidin chloride; Cyanidin]

## Data Availability

Data are contained within the article.

## Appendix A. Approximate Comparison of Chemical Constituents Identified in *L. caerulea* and *L. caerulea* ssp. Kamtschatica Varieties Obtained from Two Different Regions

**Table A1:** Chemical compounds identified from *L. caerulea* and *L. caerulea kamtschatica* in positive and negative ionization modes by HPLC-ion trap-MS/MS.

	**Class of Compounds**	**Identification**	**Formula**	**Calculated Mass**	**Observed Mass [M − H]^−^**	**Observed Mass [M + H]^+^**	**MS/MS Stage 1 Fragmentation**	**MS/MS Stage 2 Fragmentation**	**MS/MS Stage 3 Fragmentation**	**References**
**1**	**Flavone**	**Formononetin** [Biochanin B]	**C_16_H_12_O_4_**	**268.264**		**269**	256; 212; 195; 173	240; 193		*Dryopteris* [88]; *Glycyrrhiza glabra* [62]; Huolisu Oral Liquid [63]; *Dracocephalum jacutense* [64]
**2**	**Flavone**	**Apigenin** [5,7-Dixydroxy-2-(40Hydroxyphenyl)-4H-Chromen-4-One]	**C_15_H_10_O_5_**	**270.236**		**271**	243; 153	197; 161		*Inula graveolens* [43]; *Ribes meyeri* [50]; *Ribes triste* [67]; Jatropha [68]
**3**	**Flavone**	**Prunetin** [Padmakastein]	**C_16_H_12_O_5_**	**284.263**		**285**	239; 201; 185	157		*Triticum aestivum* [20]; *Zea mays* [89]; *Rosa rugosa* [69]; *Rosa amblyotis* [70]
**4**	**Flavone**	**Luteolin [Tetrahydroxyflavone]**	**C_15_H_10_O_6_**	**286.236**		**287**	271; 245; 187; 137	271		*Dryopteris* [88]; *Inula graveolens* [43]; *Ribes meyeri* [50]; *Rosa rugosa* [70]; *Rosa rugosa* [69]; Jatropha [68]
**5**	**Flavone**	**2′-Hydroxygenistein**	**C_15_H_10_O_6_**	**286.2363**		**287**	231	165		Mexican lupine species [71]
**6**	**Pentahydroxyflavone**	**Herbacetin [3,5,7,8-Tetrahydroxy-2-(4-hydro- xyphenyl)-4H-chromen-4-one]**	**C_15_H_10_O_7_**	**302.2357**		**303**	203	175		*Triticum aestivum* [20]; Ocimum [90]
**7**	**Flavone**	**Nepetin [6-Methoxyluteolin]**	**C_16_H_12_O_7_**	**316.2623**		**317**	302; 285; 229; 139	274; 153	217; 175; 153	Honey [39]; *Ribes pauciflorum* [67]
**8**	**Flavone**	**Jaceosidin** [5,7,4′-trihydroxy-6′,5’-dimethoxyflavone]	**C_17_H_14_O_7_**	**330.2889**		**331**	289	271		*Artemisia argyl* [91]; *Mentha* [92]; *Rosa acicularis* [70]
**9**	**Isoflavone**	**Sophoraisoflavone A**	**C_20_H_16_O_6_**	**352.3374**		**353**	335	317; 243; 137	191	Chinese herbal formula Jian-Pi-Yi-Shen pill [93]
**10**	**Flavone**	**Dihydroxy-tetramethoxy(iso)flavone**	**C19H18O8**	**374.3414**		**375**	345	245	175; 227	Propolis [94]
**11**	**Flavone**	**Apigenin-7-*O*-** **β-D-glucopyranoside**	**C_21_H_20_O_10_**	**432.3775**	**431**		385; 223	153		*Eucalyptus Globulus* [95]
**12**	**Flavone**	**Luteolin 7-*O*-glucoside** [Cynaroside]	**C_21_H_20_O_11_**	**448.3769**		**449**	287	213	185	*Lonicera henryi* [19]; *Lonicera japonica* [18]
**13**	**Flavone**	**Diosmetin O-hexoside**	**C_22_H_22_O_11_**	**462.4035**		**463**	301	286	258	Andean blueberry [96]
**14**	**Flavone**	**Lonicerin** [Luteolin-7-*O*-Rhamnoside; Veronicastroside; Luteolin-7-Rhamnoglucoside]	**C_27_H_30_O_15_**	**594.5181**		**595**	287; 449	269; 241; 213	213; 185	*Lonicera japonica* [18]; *Ribes triste* [67]; *Rosa rugosa* [70]
**15**	**Flavone**	**Luteolin 7-*O*-(6-*O*-rhamnosyl-hexoside)**	**C_27_H_30_O_15_**	**594.5181**		**595**	449	213	213; 157	*Lonicera henryi* [19]; *Rosa rugosa* [70]
**16**	**Isoflavone**	**Luteolin 3′,7-*O*-diglucoside**	**C_27_H_30_O_16_**	**610.5175**		**611**	449; 287	287; 199; 137	271	*Triticum aestivum* [20]; Mexican lupine species [71]; *Loropetalum chinense* [97]
**17**	**Flavone**	**Chrysoeriol O-diglucoside**	**C_28_H_32_O_16_**	**624.5441**		**625**	301; 463	286	258	Mexican lupine species [71]
**18**	**Flavonol**	**Kaempferol**	**C_15_H_10_O_6_**	**286.2363**		**287**	269; 149	239; 181		*Inula graveolens* [43]; *Juglans mandshurica* [44]; *Lonicera japonica* [18]; *Ribes meyeri* [50]; Andean blueberry [96]; *Ribes triste* [67]
**19**	**Flavonol**	**Rhamnocitrin**	**C_16_H_12_O_6_**	**300.2629**		**301**	273	245	217; 177; 131	*Astragali radix* [98]
**20**	**Flavonol**	**Quercetin**	**C_15_H_10_O_7_**	**302.2357**		**303**	257; 146	229	201; 145	*Ribes meyeri* [50]; Propolis [94]
**21**	**Flavonol**	**Hesperitin** [Hesperetin]	**C_16_H_14_O_6_**	**302.2788**		**303**	285; 165	257	229; 145	Andean blueberry [96]; *Ribes triste* [67]; *Rosa rugosa* [70]; *Rhinacanthus nasutus* [58]
**22**	**Flavonol**	**Isorhamnetin** [Isorhamnetol; Quercetin 3′-Methyl ether]	**C_16_H_12_O_7_**	**316.2623**		**318**	302; 285; 229	274	217; 175; 153	*Inula viscosa* [99]; Andean blueberry [96]; *Ribes triste* [67]; *Rosa rugosa* [70]; *Phoenix dactylifera* [100]; *Cyperus laevigatus* [101]
**23**	**Flavonol**	**Myricetin**	**C_15_H_10_O_8_**	**318.2351**		**319**	273; 219	191	209	*Juglans mandshurica* [44]; Andean blueberry [96]
**24**	**Flavonol**	**Astragalin** [Kaempferol 3-*O*-glucoside]	**C_21_H_20_O_11_**	**448.3769**		**449**	287	287; 229	203	*Juglans mandshurica* [44]; *Lonicera japonica* [18]; *Ribes meyeri* [50]; *Spondias purpurea* [102]
**25**	**Flavonol**	**Kaempferol-3-*O*-hexoside**	**C_21_H_20_O_11_**	**448.3769**		**449**	287	287; 231	230; 111	Andean blueberry [96]; *Cuphea ignea* [103]
**26**	**Flavonol**	**Quercetin-3-*O*-hexoside**	**C_21_H_20_O_12_**	**464.3763**	**463**		301	255; 229; 179; 151	185; 147	Cranberry [104]; Cherimoya; Strawberry [105]; *Rosa rugosa* [70]
**27**	**Flavonol**	**Hyperoside** (Quercetin 3-*O*-galactoside; Quercetin-3-*O*-beta-D-galactopyranoside)	**C_21_H_20_O_12_**	**464.3763**		**465**	303	285; 257; 229; 201; 165; 137	229; 201	*Dryopteris* [88]; *Lonicera japonica* [18]; Cranberry [106]; Andean blueberry [96]
**28**	**Flavonol**	**Quercetin 3-glucoside**	**C_21_H_20_O_12_**	**464.3763**	**463**		301	255; 229; 179; 151	185; 147	*Ribes meyeri* [50]; *Lonicera henryi* [19]; *Lonicera japonica* [18]; *Spondias purpurea* [102]; Andean blueberry [96]; *Rosa rugosa* [70]
**29**	**Flavonol**	**Taxifolin-3-*O*-hexoside** [Dihydroquercetin-3-*O*-hexoside]	**C_21_H_22_O_12_**	**466.3922**	**465**		285	241	198	*Rubus ulmifolius* [26]; Andean blueberry [96]; Chilean currants [25]; *Euphorbia hirta* [107]
**30**	**Flavonol**	**Quercetin 3-(6-*O*-acetyl)glucoside** [Quercetin 3-*O*-6”-acetylglucoside]	**C_23_H_22_O_13_**	**506.413**		**507**	303	285; 257; 229; 195; 165	229; 201; 173	*Malus toringoides* [108]; *Rapeseed petals* [42]
**31**	**Flavonol**	**Isorhamnetin acetylglucoside**	**C_24_H_24_O_13_**	**520.4468**		**521**	317	302; 285; 229; 165	274; 153	*Pear* [109]; *Senecio clivicolus* [65]
**32**	**Flavonol**	**Kaempferol 3-*O*-rutinoside**	**C_27_H_30_O_15_**	**594.5181**		**595**	449; 287	287	287	*Ribes meyeri* [50]; *Lonicera japonica* [18]; *Spondias purpurea* [102]
**33**	**Flavonol**	**Kaempferol glucosyl-rhamnoside**	**C_27_H_30_O_15_**	**594.5181**		**595**	449; 287	287	287	*Ribes nigrum* [110]
**34**	**Flavonol**	**Quercetin 3-*O*-pentosyl hexoside**	**C_26_H_28_O_16_**	**596.4909**		**597**	465; 303	257	229	*F. pottsii* [52]; *Spondias purpurea* [102]
**35**	**Flavonol**	**Quercetin 3-*O*-arabinoglucoside**	**C_26_H_28_O_16_**	**596.4909**		**597**	465; 303	257	229	*Spondias purpurea* [102]
**36**	**Flavonol**	**Rutin** (Quercetin 3-*O*-rutinoside)	**C_27_H_30_O_16_**	**610.5175**		**611**	303	257; 165	229	*Rubus occidentalis* [66]; *Ribes meyeri* [50]; *Lonicera henryi* [19]; *Lonicera japonica* [18]
**37**	**Flavonol**	**Kaempferol-3,7-Di-*O*-glucoside**	**C_27_H_30_O_16_**	**610.5175**		**6111**	287; 449	287; 213	213	*Taraxacum officinale* [111]; *Rapeseed petals* [42]
**38**	**Flavonol**	**Isorhamnetin 3-*O*-(6”-*O*-rhamnosyl-hexoside)**	**C_28_H_32_O_16_**	**624.5441**		**625**	317	302		*Lonicera henryi* [19]
**39**	**Flavonol**	**Kaempferol derivative**	**C_27_H_32_O_17_**	**628.5328**	**627**		465; 285	285	241	*Cuphea ignea* [103]
**40**	**Flavonol**	**Kaempferol O-acetyl hexosyl-rhamnoside**	**C_29_H_32_O_16_**	**636.5548**		**637**	287	219	171	*A. cordifolia* [52]
**41**	**Flavonol**	**Rhamnosyl-hexosyl-acyl-quercetin**	**C_29_H_30_O_17_**	**650.5383**		**651**	301; 258	286	258	*Phoenix dactylifera* [100]
**42**	**Flavonol**	**Dimethylquercetin-3-*O*-dehexoside**	**C_29_H_34_O_17_**	**654.5701**	**653**		507; 353	329		*Capsicum annuum* [112]
**43**	**Flavonol**	**Quercetin deoxyhexosyl deoxyhexosyl hexoside**	**C_33_H_40_O_20_**	**756.6587**	**755**		300; 489; 737	271	243	*Ribes meyeri* [50]
**44**	**Flavonol**	**Quercetin pentosyl hexoside hexoside**	**C_32_H_38_O_21_**	**758.6315**		**758**	670; 479; 319	415		*F. glaucescens*, *A. cordifolia* [52]
**45**	**Flavonol**	**Quercetin-3-*O-*rhamnoside derivative**	**C_42_H_40_O_22_**	**896.7538**		**897**	735	573; 447; 287	287	*Carpinus betulus* [47]
**46**	**Flavan-3-ol**	**Epiafzelechin [**(epi)Afzelechin]	**C_15_H_14_O_5_**	**274.2687**		**275**	245; 219; 175	215; 193; 175; 157; 127	175; 157; 145	*Cassia abbreviata* [113]; *F. glaucescens*; *A. cordifolia* [52]
**47**	**Flavan-3-ol**	**Catechin**	**C_15_H_14_O_6_**	**290.2681**		**291**	261; 157	191	173	*Ribes meyeri* [50]; *Ribes magellanicum* [25]
**48**	**Flavan-3-ol**	**(Epi)-catechin**	**C_15_H_14_O_6_**	**290.2681**		**291**	273; 137			*Rubus occidentalis* [66]; Cranberry [106]; *Vaccinium myrtillus* [114]; Andean blueberry [96]
**49**	**Flavan-3-ol**	**Gallocatechin**	**C_15_H_14_O_7_**	**306.2675**		**307**	289; 261; 187; 123	243; 201; 173; 135	215; 187; 145	*Carpinus betulus* [47]; *G. linguiforme*, *A. cordifolia* [52]; Ribes meyeri [50]; *Rosa rugosa* [70]; *Embelia* [54]
**50**	**Flavan-3-ol**	**(Epi)-Gallocatechin**	**C_15_H_14_O_7_**	**306.2675**		**307**	289; 261; 187	243; 173; 135	215; 187; 145	*Ribes meyeri* [50]; *Ribes magellanicum* [25]; *Vaccinium myrtillus* [114]; *Loropetalum chinense* [97]
**51**	**Flavan-3-ol**	**(Epi)-catechin derivative 1**		**379.234**		**379**	261	233	151	*PubChem*
**52**	**Flavan-3-ol**	**(Epi)-afzelechin derivative**	**C_18_H_16_O_10_**	**392.3136**		**393**	275; 245; 215	245; 175	175; 127	*Zostera marina* [115]
**53**	**Flavan-3-ol**	**(Epi)-catechin derivative 2**	**C_18_H_16_O_11_**	**408.3130**		**409**	291; 275	261; 242; 208; 173	244; 214; 191; 173; 160; 124	*PubChem*
**54**	**Flavan-3-ol**	**(Epi)-catechin derivative 3**		**424.378**		**425**	291	261; 191	191	*PubChem*
**55**	**Flavan-3-ol**	**(-)-Epicatechin Gallate** [(-)-Epicatechin-3-*O*-Gallate; L-Epicatechin Gallate]	**C_22_H_18_O_10_**	**442.3723**	**441**		330; 205	149		*G. linguiforme* [52]; *Cassia abbreviates* [113]; *Ribes meyeri* [50]; Chinese herbal formula Jian-Pi-Yi-Shen pill [93]
**56**	**Flavan-3-ol**	**(epi)Catechin O-hexoside**	**C_21_H_24_O_11_**	**452.4087**		**453**	289; 129	129	111	Andean blueberry [96]
**57**	**Flavan-3-ol**	**(Epi)gallocatechin-(epi)catechin dimer**	**C_30_H_26_O_13_**	**594.5286**		**595**	577; 409; 247	229	183	Carao tree seeds [116]
**58**	**Tannin**	**Proanthocyanidin B1** [Procyanidin Dimer B1; (-)-epicatechin-(4beta->8)-(+)-catechin]	**C_30_H_26_O_12_**	**578.5202**		**579**	409; 291	287	245	*Vaccinium myrtillus* [114]; Andean blueberry [96]; *strawberry* [29]
**59**	**Tannin**	**Proanthocyanidin B-type**	**C_30_H_26_O_13_**	**594.5196**		**595**	287; 449	213		*Actinidia chinensis* [57]
**60**	**Ellagitannin**	**Di-*O*-galloyl-HHDP-glucose** [Pedunculagin II]	**C_34_H_26_O_22_**	**786.5570**		**786**	599; 761	301		*Eugenia caryophyllata* [117]; *Cuphea ignea* [103]
**61**	**Dimeric proanthocyanidin**	**Epigallocatechin gallate dimer**	**C_44_H_34_O_22_**	**914.7276**		**915**	735; 573; 423; 329	573; 447; 287	447; 287	*Rhodiola rosea* [118]
**62**	**Anthocyanin**	**Anthocyanidin [cyanidin chloride; Cyanidin]**	**C_15_H_11_O_6+_**	**287.2442**		**287**	286; 270; 247; 205	221	201	*F. herrerae*, *G. linguiforme* [52]; Andean blueberry [96]; *Phoenix dactylifera* [100]
**63**	**Anthocyanin**	**Delphinidin**	**C_15_H_11_O_7_**	**303.2436**		**303**	257; 165	229		*A. cordifolia*, *G. linguiforme* [52]
**64**	**Anthocyanin**	**Petunidin**	**C_16_H_13_O_7+_**	**317.2702**		**318**	256	238; 113	238	*A. cordifolia*; *C. edulis*; *G. linguiforme* [52]
**65**	**Anthocyanin**	**Cyanidin-3-*O*-glucoside** [Cyanidin 3-*O*-beta-D-Glucoside]	**C_21_H_21_O_11_+**	**449.3848**		**449**	287	213	185	*Ribes magellanicum* [25]; *Berberis ilicifolia*; *Berberis empetrifolia*; *Ribes magellanicum*; *Ribes cucullatum*; *Myrteola nummalaria*; *Gaultheria mucronata*; *Gaultheria antarctica*; *Rubus geoides*; *Fuchsia magellanica* [27]
**66**	**Anthocyanin**	**Cyanidin-3-*O*-hexoside**	**C_21_H_21_O_11_+**	**449.3849**		**449**	287	213; 137	185; 157	*Dryopteris* [88]; Andean blueberry [96]; *Ribes dikuscha* [67]; *Rosa acicularis* [70]
**67**	**Anthocyanin**	**Cyanidin-3-*O*-beta-galactoside**	**C_21_H_21_O_11_**	**449.3848**		**449**	287	287; 213	185	Black soybean [21]; *Gaultheria mucronata*; *Fuchsia magellanica* [27]; *Rapeseed petals* [42]
**68**	**Anthocyanin**	**Peonidin-3-*O*-glucoside**	**C_22_H_23_O_11 +_**	**463.4114**		**463**	301	286	258	Black soybean [21]; *Berberis ilicifolia*; *Berberis empetrifolia*; *Fuchsia magellanica* [27]; Andean blueberry [96]
**69**	**Anthocyanin**	**Delphinidin 3-*O*-glucoside [Mirtillin]**	**C_21_H_21_O_12+_**	**465.3905**		**465**	303	285; 257; 229; 195; 165; 137	229; 201	*Dryopteris* [88]; *Ribes magellanicum* [25]; *Berberis ilicifolia*; *Berberis empetrifolia*; *Ribes magellanicum*; *Ribes cucullatum*; *Fuchsia magellanica* [27]; *Rosa rugosa* [70]
**70**	**Anthocyanin**	**Delphinidin 3-*O*-hexoside**	**C_21_H_21_O_12+_**	**465.3905**	**463**		301	255; 229; 179; 151	185; 147	*Dryopteris* [88]; Andean blueberry [96]; *Rosa rugosa* [70]
**71**	**Anthocyanin**	**Delphinidin 3-*O-beta-*galactoside**	**C_21_H_21_O_12+_**	**465.3905**		**465**	303	285; 257; 229; 165; 137	229; 201	*Dryopteris* [88]; *Gaultheria micronata*; *Gaultheria antarctica*; *Fuchsia magellanica* [27]; *Rosa rugosa* [70]
**72**	**Anthocyanin**	**Delphinidin 3-acetylglucoside** [Delphinidin-3-*O*-(6-*O*-acetyl)glucoside]	**C_23_H_23_O_13+_**	**507.4209**		**507**	303	257	229	*Vitis vinifera* [31]; Vines [32]; Grape [40]
**73**	**Anthocyanin**	**Delphinidin-*3-O-(6”-O*-acetyl)hexoside**	**C_23_H_23_O_13+_**	**507.4209**		**507**	303	285; 257; 229; 195	229; 201; 173	*Vitis vinifera* [30]
**74**	**Anthocyanin**	**Petunidin 3-*O*-(6-*O*-acetyl)glucoside**	**C_24_H_25_O_13_**	**521.451**		**521**	317	302	274	*Vitis vinifera* [31]; Vines [32]; Grape [40]
**75**	**Anthocyanin**	**Cyanidin-3-*O*-rutinoside** [Keracyanin; Antirrhinin; Sambucin]	**C_27_H_31_O_15_**	**595.526**		**595**	287; 449	213		*Ribes magellanicum* [25]; *Berberis microphylla* [28]; *Berberis ilicifolia*; *Berberis empetrifolia*; *Ribes magellanicum*; *Ribes cucullatum* [27]; *Ribes triste* [67]
**76**	**Anthocyanin**	**Delphinidin 3-*O*-Beta-*D*-sambubioside**	**C_26_H_29_O_16_**	**597.4989**		**597**	303; 465; 229	229; 165	201; 172	*Berberis microphylla* [28]; *Red currant* [24]; *Ribes dikuscha*; *Ribes triste* [67]
**77**	**Anthocyanin**	**Delphinidin 3-*O*-[2-*O*-(Beta-xylosyl)-beta-galactoside]**	**C_26_H_29_O_16_**	**597.4989**		**597**	303	257; 165	229; 201; 161	*Red Kiwifruit* [23]
**78**	**Anthocyanin**	**Peonidin 3-*O*-rutinoside**	**C_28_H_33_O_15_**	**609.5526**		**609**	463; 301	286		*Gaultheria mucronata*; *Gaultheria antarctica*; *Fuchsia magellanica* [27]; *Black currant*, *Gooseberry* [24]; *Aristotelia chilensis* [119]
**79**	**Anthocyanin**	**Peonidin 3-*O*-(6-*O-p-*coumaroyl)glucoside**	**C_31_H_29_O_13_**	**609.554**		**609**	301; 542	258		Grape [40] Vines [32]; *Vitis vinifera* [31]
**80**	**Anthocyanin**	**Delphinidin 3-*O*-rutinoside [Tulipanin; Delphinidin 3-Rhamnosyl-Glucoside]**	**C_27_H_31_O_16_**	**611.5254**		**611**	465; 303	229	201	*Dryopteris* [88]; *Vigna angularis* [120]; Black *currant* [24]; *Berberis ilicifolia*; *Berberis empetrifolia*; *Ribes magellanicum*; *Fuchsia magellanica* [27]; *Berberis microphylla* [28]
**81**	**Anthocyanin**	**Delphinidin 3-*O*-(6-*O-p-*coumaroyl) glucoside**	**C_30_H_27_O_14_**	**611.5270**		**611**	465; 303	229	201	*Dryopteris* [88]; *Vigna angularis* [120]; Grape [40] *Vitis vinifera* [31]; Vines [32]; *Ribes triste* [67]
**82**	**Anthocyanin**	**Cyanidin 3,5-*O*-diglucoside**	**C_27_H_31_O_16_**	**611.5335**		**611**	287; 449	213	185	Grape [40]; Muscadine pomace [41]; *Berberis microphylla* [28]; *Rapeseed petals* [42]; *Lonicera caerulea* [16]
**83**	**Hydroxycinnamic acid**	**4-Hydroxybenzoic acid** [PHBA; Benzoic acid; p-Hydroxybenzoic acid]	**C_7_H_6_O_3_**	**138.1207**		**139**	111			*Inula graveolens* [43]; Ocimum [90]; *Taraxacum formosanum* [121]; *Bituminaria* [122]; Eucalyptus Globulus [95]
**84**	**Hydroxybenzoic acid (Phenolic acid)**	**Protocatechuic acid**	**C_7_H_6_O_4_**	**154.1201**		**155**	127			*Ribes meyeri* [50]; *Lonicera japonica* [18]
**85**	**Hydroxycinnamic acid**	**3,4-Dihydroxyhydrocinnamic acid [Dihydrocaffeic acid]**	**C_9_H_10_O_4_**	**182.1733**		**183**	155	127	**145**	*Eucalyptus Globulus* [95]; Chilean currants [25]
**86**	**Dihydroxybenzoic acid**	**Ethyl protocatechuate [3,4-Dihydroxybenzoic Acid Ethyl Ester]**	**C_9_H_10_O_4_**	**182.1733**		**183**	155	111		*Dryopteris* [88]; Ocimum [90]
**87**	**Phenolic acid**	**Methylgallic acid [Methyl gallate; Methyl 3,4,5-trihydroxybenzoate]**	**C_8_H_8_O_5_**	**184.1461**		**185**	155; 127	127	**117**	*Papaya* [105]; Andean blueberry [96]; *Ribes triste* [67]; Phyllanthus [123]
**88**	**Phenolic acid**	**Hydroxy methoxy dimethylbenzoic acid**	**C_10_H_12_O_4_**	**196.1999**		**197**	160	151		*F. herrerae*; *F. glaucescens*, *G. linguiforme* [52]
**89**	**Phenolic acid**	**2,3,4,5,6-pentahydroxybenzoic acid**	**C_7_H_6_O_7_**	**202.1183**		**203**	156	129		*Jatropha* [68]
**90**	**Hydroxycinnamic acid**	**Hydroxyferulic acid**	**C_10_H_10_O_5_**	**210.1834**		**211**	193	75; 147	157; 129	Andean blueberry [96]; *Rosa davurica* [124]
**91**	**Hydroxybenzoic acid (Phenolic acid)**	**Ellagic acid** [Benzoaric acid; Elagostasine]	**C_14_H_6_O_8_**	**302.1926**	**301**		257	229	201	*Juglans mandshurica* [44]; *Eucalyptus* [125]; *Eucalyptus Globulus* [95]
**92**	**Phenolic acid**	**p-Coumaroylquinic acid**	**C_16_H_18_O_8_**	**338.3093**		**339**	321; 121	320		*Vaccinium myrtillus* [114]; Andean blueberry [96]; *Ribes magellanicum* [25]; *Ribes meyeri* [50]
**93**	**Hydroxycinnamic acid;**	**Chlorogenic acid** [3-*O*-Caffeoylquinic acid]	**C_16_H_18_O_9_**	**354.3087**	**353**		191	127		*Ribes magellanicum* [25]; *Lonicera henryi* [19]; Cranberry [106]; *Lonicera japonica* [18]; *Vaccinium myrtillus* [114]; *Spondias purpurea* [102]; Andean blueberry [96]
**94**	**Hydroxycinnamic acid**	**Neochlorogenic acid** [5-*O*-Caffeoylquinic acid]	**C_16_H_18_O_9_**	**354.3088**	**353**		191	173	126	*Lonicera henryi* [19]; *Ribes magellanicum* [25]; *Lonicera japonica* [18]; *Vaccinium myrtillus* [114]; Andean blueberry [96]
**95**	**Hydroxycinnamic acid;**	**3-*O*-Hydroxydihydrocaffeoylquinic acid**	**C_16_H_20_O_10_**	**372.3240**	**371**		191	173; 127		*Lonicera henryi* [19]
**96**	**Phenolic acid**	**Caffeoylquinic acid derivative**		**382.6633**	**381**		179; 135	135		*Vaccinium myrtillus* [114]
**97**	**Phenolic acid**	**Ferulic acid-*O*-hexoside derivative**	**C_21_H_22_O_11_**	**450.3928**	**449**		269; 151	225; 151		strawberry [29]; *Cuphea ignea* [103]
**98**	**Phenolic acid**	**Ellagic acid-*O*-hexoside**	**C_20_H_16_O_13_**	**464.3332**	**463**		301	255; 229; 179; 151	185; 147	*Carpinus betulus* [47]; *Punica granatum* [126]
**99**	**Phenolic acid**	**Dicaffeoyl shiikimic acid**	**C_25_H_22_O_11_**	**498.4356**		**499**	163; 319	145	117	Andean blueberry [96]
**100**	**Phenolic acid**	**p-Coumaroyl malonyldihexose**		**574.8314**		**575**	413; 335; 188	395; 340; 226; 188	346; 290; 211	*Vaccinium myrtillus* [114]
**101**	**Phenolic acid**	**Feruloyl-*O*-p-coumaroyl-*O*-caffeoylshikimic acid**		**676**		**677**	513; 367	349; 266		*Phoenix dactylifera* [100]
**102**		**Caffeic acid isoprenyl ester**	**C_14_H_16_O_4_**	**248.2744**		**249**	203; 157	157	129	*Eucalyptus* [125]; Brazilian propolis [127]; *Ribes triste* [67]
**103**	**Dihydrochalcone**	**Phloretin** [Dihydronaringenin]	**C_15_H_14_O_5_**	**274.2687**		**275**	257	230	229	*Malus toringoides* [108]; *G. linguiforme* [52]; *Punica granatum* [126]
**104**	**Stilbene**	**Pinosylvin** [ 3,5-Stilbenediol; Trans-3,5-Dihydroxystilbene]	**C_14_H_12_O_2_**	**212.2439**		**213**	167; 139	139		*Pinus resinosa* [128]; *Pinus sylvestris* [129]
**105**	**Stilbene**	**Resveratrol** [trans-Resveratrol; 3,4′,5-Trihydroxystilbene; Stilbentriol]	**C_14_H_12_O_3_**	**228.2433**		**229**	211	183; 127	138	*Embelia* [54]; *A. cordifolia*; *F. glaucescens*; *G. linguiforme* [52];
**106**	**Hydroxycoumarin**	**Esculetin** [3,7-Dihydroxylcoumarin; Cichorigenin; Aesculetin]	**C_9_H_6_O_4_**	**178.1415**		**179**	151	122		*Actinidia chinensis* [57]; *Rhinacanthus nasutus* [58]; *Basilic*; *Rosemary*; *Salvia*; *Thymus vulgaris* [59]
**107**	**Coumarin**	**4-Methylesculetin** [4-Methyl-6,7-Dihydroxycoumarin]	**C_10_H_8_O_4_**	**192.1681**		**193**	147	129	110	*Artemisia annua* [130]
**108**	**Coumarin**	**Fraxetin**	**C_10_H_8_O_5_**	**208.1675**		**209**	191	117		*Embelia* [54]; *Actinidia chinensis* [57]; *Jatropha* [68]
**109**	**Isocoumarin**	**Coriandrone**	**C_13_H_10_O_5_**	**246.2155**		**247**	230; 201; 173; 145	145		*Ventilago denticulata* [131]
**110**	**Lignan**	**Syringaresinol**	**C_22_H_26_O_8_**	**418.4436**		**419**	255	239	211	Magnolia [132]; *Triticum aestivum* [133]
	**Others**									
**111**	**Aliphatic amino acid**	**L-Threonine** [(2S, 3R)-2-Amino-3-Hydroxybutanoic acid]	**C_4_H_9_NO_3_**	**119.1192**		**120**				*Triticum aestivum* [134]; *Medicago truncatula* [135]; Soybean [136]; Soybean leaves [137]; *Lonicera japonica* [18]
**112**	**Non-proteinogenic L-alpha-amino acid**	**L-Pyroglutamic acid** [Pidolic acid; 5-Oxo-L-Proline]	**C_5_H_7_NO_3_**	**129.1140**		**130**	111			*Potato leaves* [46]; Huolisu Oral Liquid [63]; *Lonicera caerulea* [16]; *Ribes pauciflorum* [67]
**113**	**Organic acid**	**Malic acid** [DL-Malic acid]	**C_4_H_6_O_5_**	**134.0874**	**133**		115			*Inula graveolens* [43]; *Punica granatum* [126]; Ribes meyeri [50]; *Ribes pauciflorum* [67]
**114**	**Aliphatic amino acid**	**L-glutamic acid; GLUTAMIC ACID; Glutaminol**	**C_5_H_10_N_2_O_3_**	**146.1445**		**147**	130			*Triticum aestivum* [134]; *Soybean leaves* [137]; *Lonicera japonica* [18]; *Rosa acicularis* [70]
**115**	**Phenylethanoid**	**3,4-Dihydroxyphenylethanol** [Hydroxytyrosol]	**C_8_H_10_O_3_**	**154.1632**		**155**	127	144		Grape [40]; *G. linguiforme* [52]
**116**	**Amino acid**	**L-Histidine**	**C_6_H_9_N_3_O_2_**	**155.1546**		**156**	129	110		*Lonicera japonica* [18]; *Actinidia deliciosa* [138]
**117**	**Indole alkaloid**	**Indole-3-acetonitrile**	**C_10_H_8_N_2_**	**156.1839**		**157**	129; 115	129; 120		Honey [39]; *Ribes pauciflorum* [67]
**118**	**Aromatic amino acid**	**Phenylalanine** [L-Phenylalanine]	**C_9_H_11_NO_2_**	**165.1891**		**166**	120			*Lonicera japonica* [18]; *Potato leaves* [46]; *G. linguiforme* [52]; *Rapeseed petals* [42]; Xuefu Zhuyu decoction [139]
**119**	**Cyclohexenecarboxylic acid**	**Shikimic acid** [L-Schikimic acid]	**C_7_H_10_O_5_**	**174.1513**		**175**	129	111		*A. cordifolia*; *G. linguiforme* [52]; *Ribes meyeri* [50]; *Ribes aureum*; *Ribes dikuscha* [67]
**120**	**Omega-hydroxy amino acid**	**Hydroxy decenoic acid**	**C_10_H_18_O_3_**	**186.2481**		**187**	145	127		*F. glaucescens*, *G. linguiforme* [52]
**121**	**Naphthoquinone**	**2,5-Dihydroxy-1,4-naphthalenedione**	**C_10_H_6_O_4_**	**190.1522**		**191**	145; 117			*Juglans mandshurica* [44]; *Ribes triste* [67]
**122**	**Naphthoquinone**	**3,5-Dihydroxy-1,4-naphthalenedione**	**C_10_H_6_O_4_**	**190.1522**		**191**	145; 127; 117			*Juglans mandshurica* [44]; *Ribes triste* [67]
**123**	**Tricarboxylic acid**	**Citric acid**	**C_6_H_8_O_7_**	**192.1235**	**191**		111; 173			*Potato leaves* [46]; *Strawberry*, *Lemon*, *Cherimoya*, *Papaya*, *Passion fruit* [105]
**124**	**Polyhydroxycarboxylic acid**	**Quinic acid**	**C_7_H_12_O_6_**	**192.1666**	**191**		111; 173	111		*Ribes meyeri* [50]; *Lonicera japonica* [18]; Andean blueberry [96]; *Potato leaves* [46]
**125**	**Alpha, omega dicarboxylic acid**	**Sebacic acid**	**C_10_H_18_O_4_**	**202.2475**		**203**	185	139	111; 157	*Jatropha* [68]
**126**	**Polysaccharides**	**Galactaric acid** [Mucic acid; Galactarate]	**C_6_H_10_O_8_**	**210.1388**		**211**	193	175	129	*Dryopteris* [88]; Soybean [136]; *Rosa rugosa* [70]
**127**	**Saturated fatty acid**	**Hydroxydodecenoic acid**	**C_12_H_22_O_3_**	**214.3013**		**215**	208	186		*Jatropha* [68]
**128**	**Sesquiterpenoid**	**Caryophyllene oxide** [Caryophyllene-alpha-oxide]	**C_15_H_24_O**	**220.3505**	**219**		173; 111	111		Olive leaves [140]; *Rosa davurica* [124]
**129**	**Omega-5 fatty acid**	**Myristoleic acid** [Cis-9-Tetradecanoic acid]	**C_14_H_26_O_2_**	**226.3550**		**227**	209	192; 139	122	*G. linguiforme* [52]; *Artemisia martjanovii* [141]
**130**	**Sesquiterpenoid**	**Atractylenolide II** [2-Atractylenolide]	**C_15_H_20_O_2_**	**232.3181**		**233**	216	160	132	*Codonopsis Radix* [142]; Chinese herbal formula Jian-Pi-Yi-Shen pill [93]
**131**	**Medium-chain fatty acid**	**Hydroxy dodecanoic acid**	**C_12_H_22_O_5_**	**246.3001**		**247**	229; 201	187	159; 145	*F. glaucescens*; *G. linguiforme* [52]
**132**	**Naphthoquinone**	**1,4-Dihydroxy-2,3-naphthalenedicarboxylic acid/6-Acetyl-2,5,8-trihydroxynaphthoquinone**	**C_12_H_8_O_6_**	**248.1883**		**249**	203	157	129	*Juglans mandshurica* [44]; *Ribes triste* [67]
**133**	**Polyunsaturated long-chain fatty acid**	**Hexadecadienoic acid**	**C_16_H_28_O_2_**	**252.3923**		**253**	145; 244	127		*Rhus coriaria* [45]
**134**	**Naphthoquinone**	**1,3,6,8-Tetrahydroxy-9(10H)-anthracenone**	**C_14_H_10_O_5_**	**258.2262**		**259**	231; 211; 200; 187; 169; 149; 127	213; 185; 164		*Juglans mandshurica* [44]; *Ribes pauciflorum* [67]
**135**	**Ribonucleoside composite of adenine (purine)**	**Adenosine**	**C_10_H_13_N_5_O_4_**	**267.2413**		**268**	136			*Lonicera japonica* [18]; Huolisu Oral Liquid [63]; *Rosa rugosa* [70]
**136**	**Omega-3 fatty acid**	**Stearidonic acid** [6,9,12,15-Octadecatetraenoic acid]	**C_18_H_28_O_2_**	**276.4137**		**278**	261; 172; 115	127		*Dryopteris* [88]; *G. linguiforme* [52]; *Rhus coriaria* [45]; *Jatropha* [68]; *Ribes triste* [67]; *Rosa rugosa* [70]
**137**	**Phenanthraquinone**	**Tanshinone V**	**C_19_H_22_O_4_**	**314.3765**	**313**		212; 113	113; 185		Huolisu Oral Liquid [63]
**138**	**Oxylipin**	**13-Trihydroxy-Octadecenoic acid [THODE]**	**C_18_H_34_O_5_**	**330.4596**	**329**		229; 211; 171; 139	211; 183; 161; 143		*Bituminaria* [122]; *Jatropha* [68]; *Phoenix dactylifera* [100]; *Rosa amblyotis* [70]; *Apium graveolens* [143]
**139**	**Cyclopentapyran; Iridoid**	**Loganic acid**	**C_16_H_24_O_10_**	**376.3558**	**375**		**213**	169; 113		*Lonicera japonica* [18]; *Rosa amblyotis* [70]
**140**	**Tricarboxylic acid**	**Citric acid derivative**		**392**	**391**		373; 217	h143		*Punica granatum* [126]
**141**	**Anabolic steroid**	**Vebonol**	**C_30_H_44_O_3_**	**452.6686**		**453**	435; 210	226; 336	210	*Rhus coriaria* [45]; *Hylosereus polyrhizus* [144]
**142**	**Thromboxane receptor antagonist**	**Vapiprost**	**C_30_H_39_NO_4_**	**477.6350**		**478**	337	263; 121	119	*Rhus coriaria* [45]; *Hylosereus polyrhizus* [144]
**143**	**Indole sesquiterpene alkaloid**	**Sespendole**	**C_33_H_45_NO_4_**	**519.7147**		**520**	184	125		*Rhus coriaria* [45]
**144**	**Chlorophyll degradation product**	**Pheophorbide a**	**C_35_H_36_N_4_O_5_**	**592.6841**						[145]
